# Possible correlation between increased serum free carnitine levels and increased skeletal muscle mass following HCV eradication by direct acting antivirals

**DOI:** 10.1038/s41598-021-96203-z

**Published:** 2021-08-16

**Authors:** Yoshimasa Tokuchi, Goki Suda, Megumi Kimura, Osamu Maehara, Takashi Kitagataya, Akinori Kubo, Sonoe Yoshida, Qingjie Fu, Zijian Yang, Shunichi Hosoda, Masatsugu Ohara, Ren Yamada, Kazuharu Suzuki, Naoki Kawagishi, Masato Nakai, Takuya Sho, Mitsuteru Natsuizaka, Kenichi Morikawa, Koji Ogawa, Shunsuke Ohnishi, Naoya Sakamoto

**Affiliations:** 1 Department of Gastroenterology and Hepatology, Hokkaido University Graduate School of Medicine, North 15, West 7, Kita-ku, Sapporo, Hokkaido 060-8638 Japan; 2 Department of Pathophysiology and Therapeutics, Faculty of Pharmaceutical Sciences, Hokkaido University, Sapporo, Japan

**Keywords:** Gastroenterology, Hepatology

## Abstract

We aimed to evaluate factors associated with changes in skeletal muscle mass in hepatitis C virus (HCV)-infected patients after treatment with direct-acting antivirals (DAAs). Consecutive HCV-infected patients after treatment with DAA were recruited into the study. Patients who achieved sustained virological response (SVR); and had complete clinical information, preserved serum samples at baseline and SVR48, and skeletal muscle mass evaluations based on the psoas muscle mass index (PMI) on computed tomography at baseline and ≥ 12 months were included. Altogether, 70.7% of patients (41/58) showed increased PMI after DAA therapy, and mean relative PMI was significantly higher after DAA therapy than at baseline. There were no significant associations between baseline clinical factors routinely examined in clinical practice and increased PMI. Among factors reported to be associated with skeletal muscle loss in patients with chronic liver disease, serum zinc levels and total and free carnitine levels increased significantly after DAA therapy and only changes in serum free carnitine levels were significantly associated with an increased PMI (*r* = 0305, *P* = 0.020). In conclusion, increased skeletal muscle mass after successful HCV eradication by DAAs was significantly associated with increased serum-free carnitine levels. l-carnitine supplementation may be beneficial in patients with low skeletal muscle mass after DAA.

## Introduction

Hepatitis C virus (HCV) infection is a major cause of hepatocellular carcinoma (HCC) and liver transplantation^1^. Recently developed direct-acting antiviral (DAA) agents have been reported to result in a sustained viral response (SVR) rate of greater than 90% in patients with HCV infection, including those with decompensated liver cirrhosis, hemodialysis, and advanced age^2–5^. Successful HCV eradication by DAAs could decrease HCC occurrence^6^ and liver fibrosis^7,8^ and restore liver function^3^. Additionally, extrahepatic dysfunction, including cryoglobulinemia and abnormal lipid profiles^9^, could be restored after successful HCV eradication by DAAs. Regarding extrahepatic dysfunction, it has been reported that successful HCV eradication by DAAs could restore skeletal muscle mass loss^10,11^.

Primary sarcopenia is defined as muscle atrophy and age-related decrease in muscle strength^12^. Chronic liver disease, including liver cirrhosis (LC) and HCC, is one of the major causes of secondary sarcopenia^12–16^. Chronic liver disease with complicating sarcopenia has poor prognosis^17^. Thus, preventing and managing this loss of skeletal muscle mass in patients with chronic liver disease are clinically important.

There is evidence that skeletal muscle mass significantly increased or skeletal muscle loss could be suppressed after successful HCV eradication. However, some patients with low skeletal muscle mass have not experienced increases in skeletal muscle, even after successful HCV eradication Thus, clarifying the mechanisms underlying changes in skeletal mass has important clinical implications. However, predictive factors and molecular mechanisms underlying this increase in skeletal muscle mass after successful HCV eradication have not been determined yet^10,11^. In this study, we evaluated factors associated with increased skeletal muscle mass after successful HCV eradication by DAAs.

## Results

### Patients

HCV-infected patients receiving interferon (IFN)-free DAA regimen between October 2014 and November 2019 at Hokkaido University Hospital were screened. Out of these, 58 patients met the inclusion criteria and were included in the study.

The baseline characteristics of patients with HCV infection are shown in Table 1. The median age of patients was 74 years (range 43–90 years), and 48.2% of them (28/58) were men. The most common HCV genotype was genotype 1 (81.0%, 47/58). The number of patients treated with daclatasvir/asunaprevir, sofosbuvir/ledipasvir, sofosbuvir/ribavirin, ombitasvir/paritaprevir/ritonavir, or other agents was 13, 19, 9, 6, and 11, respectively. A total of 44.8% (26/58) had LC, and the median fibrosis (FIB)-4 index was 5.1 (range 0.7–12.0). The baseline median PMI value was 4.22 cm^2^/m^2^ (range 2.08–8.46) in all patients: 5.30 cm^2^/m^2^ (range 2.73–8.46) in men, and 3.89 cm^2^/m^2^ (range 2.08–6.50) in women.

**Table 1:** Comparison between patients with or without increased PMI after successful HCV eradication by DAAs.

	All patients	PMI increase	PMI decrease	*P*-value
Number	n = 58	n = 41	n = 17	
Age (years)^a^	74 (43–90)	74.0 (43–90)	68.0 (45–89)	0.2573
Sex (male/female)	28/30	21/20	7/10	0.8396
Body weight (kg)	59.0 (32.0–102.0)	57.0 (45–102)	60.4 (32.0–83.0)	0.6313
BMI(kg/m^2^)	22.5 (15.0–34.1)	22.5 (19.2–34.1)	23.6 (15.0–28.6)	0.7885
HCV-RNA (log IU/mL)^a^	6.2 (3.7–7.2)	6.2 (3.7–7.1)	5.8 (3.8–7.0)	0.1861
HCV Serotype (1/2/3/4)	47/10/0/1	33/7/0/1	14/3/0/0	0.8097
DCV/ASV, SOF/LDV, SOF/RBV, OBV/PTV/r, others	13/19/9/6/11	8/15/6/3/9	5/4/3/3/2	0.5383
LC/non-LC	28/30	19/22	9/8	0.7749
Platelet count (× 10^4^)^a^	11.8 (4.3–35.9)	12.4 (4.3–27.6)	9.6 (4.7–35.9)	0.5354
Albumin (g/dL)^a^	3.7 (2.7–4.8)	3.7 (2.7–4.7)	3.6 (2.7–4.8)	0.8183
AST (IU/L)^a^	47 (17–155)	44 (23–155)	58 (17–94)	0.8254
ALT (IU/L)^a^	35 (14–128)	35 (14–114)	40 (14–128)	0.5550
FIB-4 index^a^	5.1 (0.70–12.0)	4.0 (0.94–12.0)	6.35 (0.70–9.68)	0.4283
AFP (ng/mL)^a^	6.7 (1.5–83.9)	6.6 (1.5–60.7)	9.6 (1.8–83.9)	0.1170
Creatine (mg/dL)	0.78 (0.41–1.81)	0.79 (0.41–1.81)	0.7 (0.47–1.81)	0.8030
Diabetes mellitus n (%)	11 (19.0)	6 (14.6)	5 (29.4)	0.2704
Alcohol consumption n (%)^b^	4 (6.9)	3 (7.3)	1 (5.9)	> 0.9999
Previous treatment (yes/no)	19/39	14/27	5/12	> 0.9999
PMI (cm^2^/m^2^)^a^	4.22 (2.08–8.46)	4.19 (2.08–8.46)	4.50 (2.49–8.31)	0.5936
Male	5.30 (2.73–8.46)	5.27 (2.73–8.46)	5.28 (2.89–8.31)	0.7124
Female	3.89 (2.08–6.50)	3.77 (2.08–6.50)	4.22 (2.49– .52)	0.3069

*HCV* hepatitis C virus, *DAAs* direct acting antivirals, *AST* aspartate aminotransferase, *ALT* alanine aminotransferase, *γGTP* γ-glutamyl transpeptidase, *FIB-4* fibrosis 4, *AFP* alpha-fetoprotein tumor marker, *DCV* daclatasvir, *ASV* asunaprevir, *SOF* sofosbuvir, *LDV* ledipasvir, *RBV* ribavirin, *OBV* ombitasvir, *PTV* paritaprevir.

^a^Data are shown as median (range) values.

^b^Alcohol intake > 20 g/day for women and > 30 g/day for men.

*Significant difference, *P* < 0.05.

### Changes in skeletal muscle mass and related factors after successful HCV eradication by DAAs

**Figure 1:**
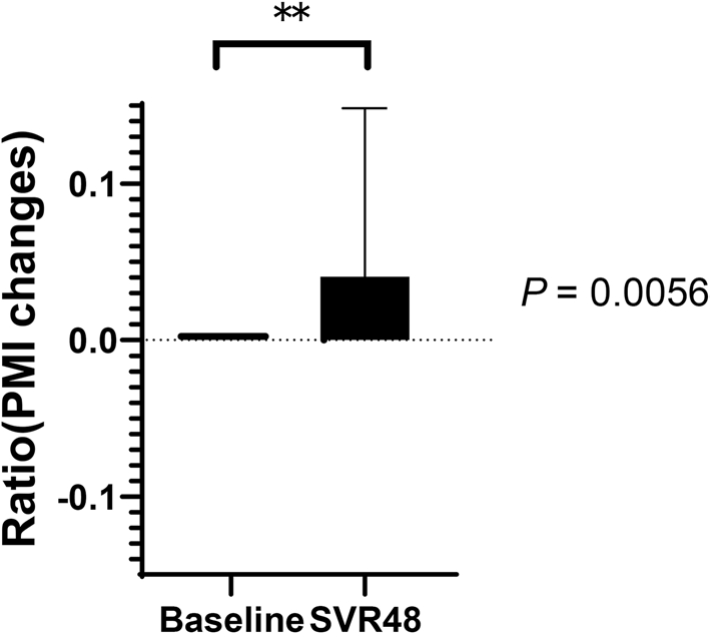
Relative changes in skeletal muscle mass after successful HCV eradication by direct-acting antiviral agents (DAAs) at SVR48. SVR48; sustained virological response at 48 weeks after treatment completion. *PMI* psoas muscle mass index. Data are shown as means ± standard deviation (SD). Asterisks indicate a statistically significant difference (**P < 0.01).

As shown in Table 1, 70.7% of patients (41/58) experienced increased PMI after DAA therapy. In addition, the relative PMI values were significantly higher after DAA treatment than those at baseline (*P* = 0.0056) (Fig. 1). Table 1 shows a comparison of patients with or without increased PMI after successful HCV eradication by DAAs. No factors that were routinely evaluated in the clinical practice setting differed significantly between patients with or without increased PMI after successful HCV eradication by DAAs.

We further analyzed factors associated with changes in skeletal muscle mass in patients with chronic liver disease. We analyzed serum vitamin D; insulin-like growth factor 1 (IGF-1); zinc, branched chain amino acid (BCAA); and total and free carnitine levels. These parameters were previously reported to be associated with skeletal muscle loss in patients with chronic liver disease^18,19^, at baseline and SVR48.

**Figure 2:**
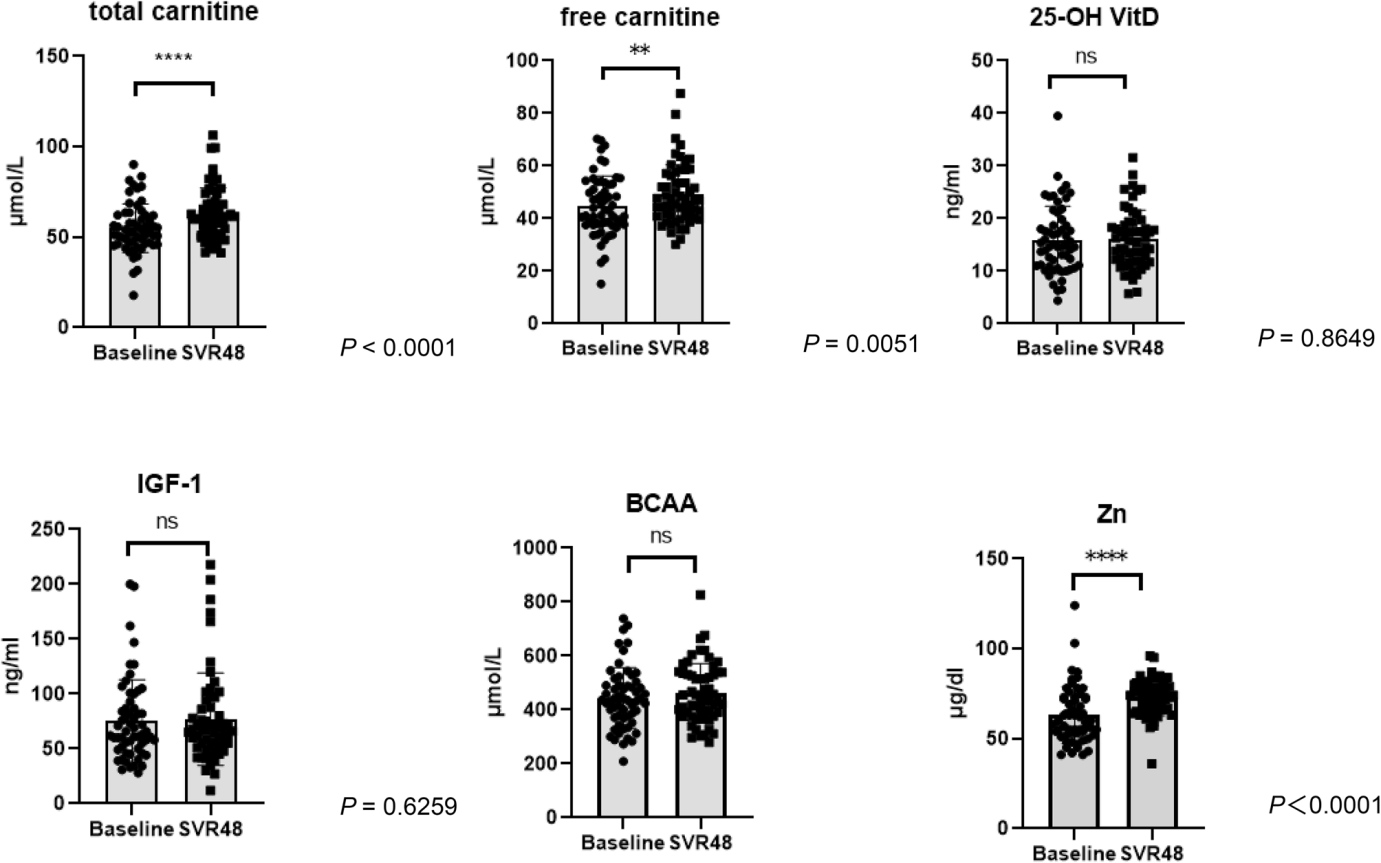
Comparison of serum vitamin D, IGF-1, zinc, BCAA, and total and free carnitine levels between baseline and at SVR48. SVR48; sustained virological response at 48 weeks after the completion of treatment. *IGF1* insulin-like growth factor-1, *BCAA* branched chain amino acid, *25-OH vitamin D* 25-hydroxyvitamin D, *PMI* psoas muscle mass index. Data are shown as mean ± standard deviation (SD). Asterisks indicate statistically significant differences (**P < 0.01, ****P < 0.0001).

**Figure 3:**
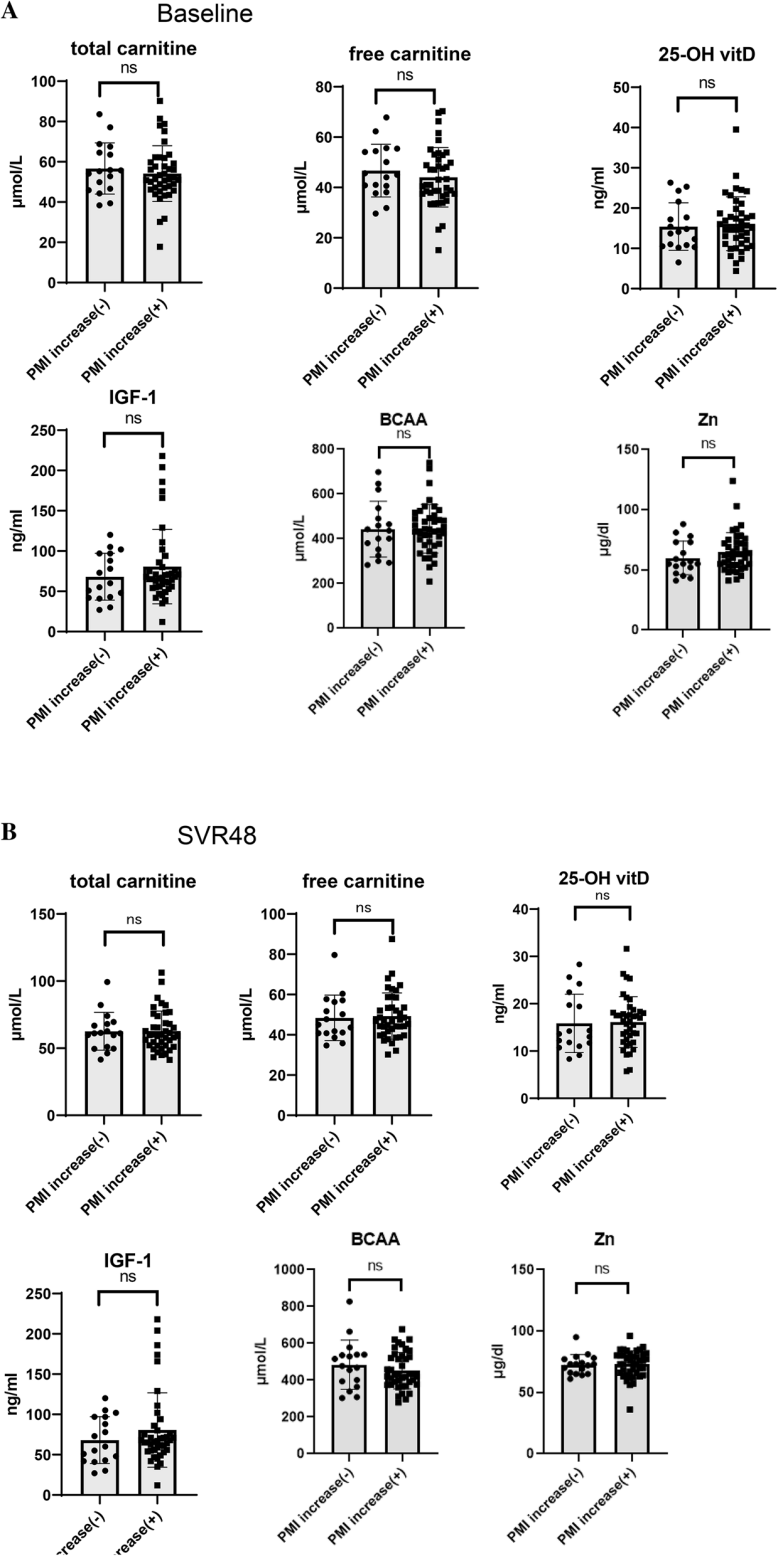
Comparison of serum vitamin D, IGF-1, zinc, BCAA, and total and free carnitine levels at baseline or SVR 48 between patients with and without PMI increases. (**a**) Comparison of serum vitamin D, IGF-1, zinc, BCAA, and total and free carnitine levels at baseline between patients with and without a PMI increase. (**b**) Comparison of serum vitamin D, IGF-1, zinc, BCAA, and total and free carnitine levels at SVR48 between patients with and without a PMI increase. *SVR48* sustained virological response at 48 weeks after the completion of treatment. *IGF1* insulin-like growth factor-1, *BCAA* branch chain amino acid, *25-OH vitamin D* 25-hydroxyvitamin D, *PMI* psoas muscle mass index. Data are shown as mean ± standard deviation (SD). Asterisk indicate statistically significant differences (**P < 0.01, ****P < 0.0001).

As shown in Fig. 2, levels of zinc (*P* < 0.0001), total carnitine (*P* < 0.0001), and free carnitine (*P* = 0.0051) were significantly higher after successful HCV eradication than those at baseline. Serum 25-hydroxyvitamin D (25(OH)-vitamin D), Insulin-like growth factor 1(IGF-1), and BCAA levels did not change after DAA therapy. Subsequently, we compared the levels at baseline and at the time that SVR was achieved between patients with or without PMI after successful HCV eradication. As shown in Fig. 3a,b, the levels of 25(OH)-vitamin D, IGF-1, BCAA, zinc, total carnitine, and free carnitine were similar between patients with or without PMI increase at baseline and SVR48.

**Figure 4:**
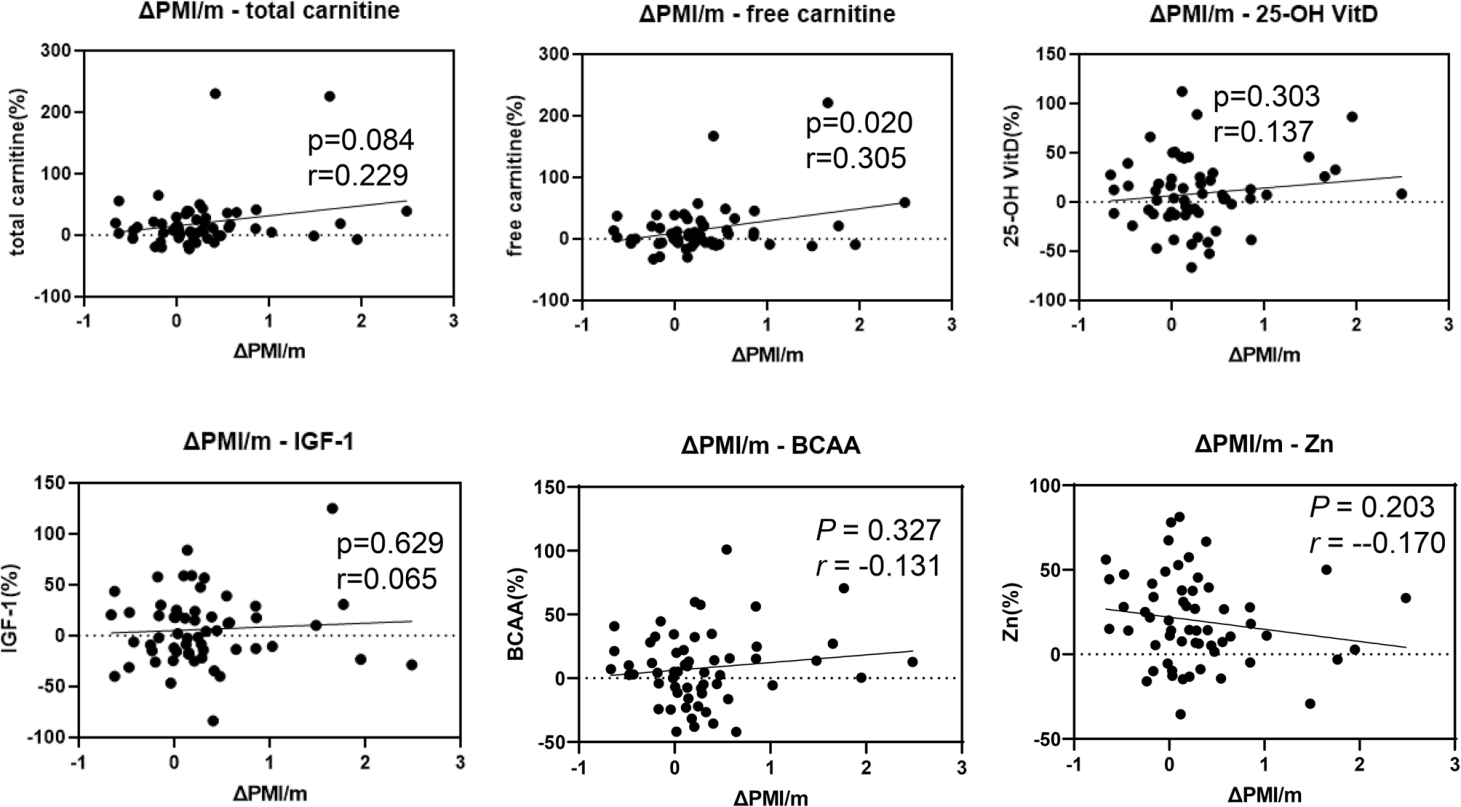
Correlations between changes in the psoas muscle mass index (PMI)/month (ΔPMI/month) and changes in serum total/free creatinine, vitamin D, IGF-1, BCAA, and zinc. *IGF1* insulin-like growth factor-1, *BCAA* branch chain amino acid, *25(OH)-vitamin D* 25-hydroxyvitamin D, *PMI* psoas muscle mass index. Data were analyzed using Spearman’s rank correlation.

Subsequently, we analyzed correlations between ΔPMI/month and serum total/free creatinine, vitamin D, IGF-1, BCAA, and zinc levels. As shown in Fig. 4, only changes in free carnitine were significantly correlated with ΔPMI/month (*r* = 0.305, *P* = 0.025).

**Figure 5:**
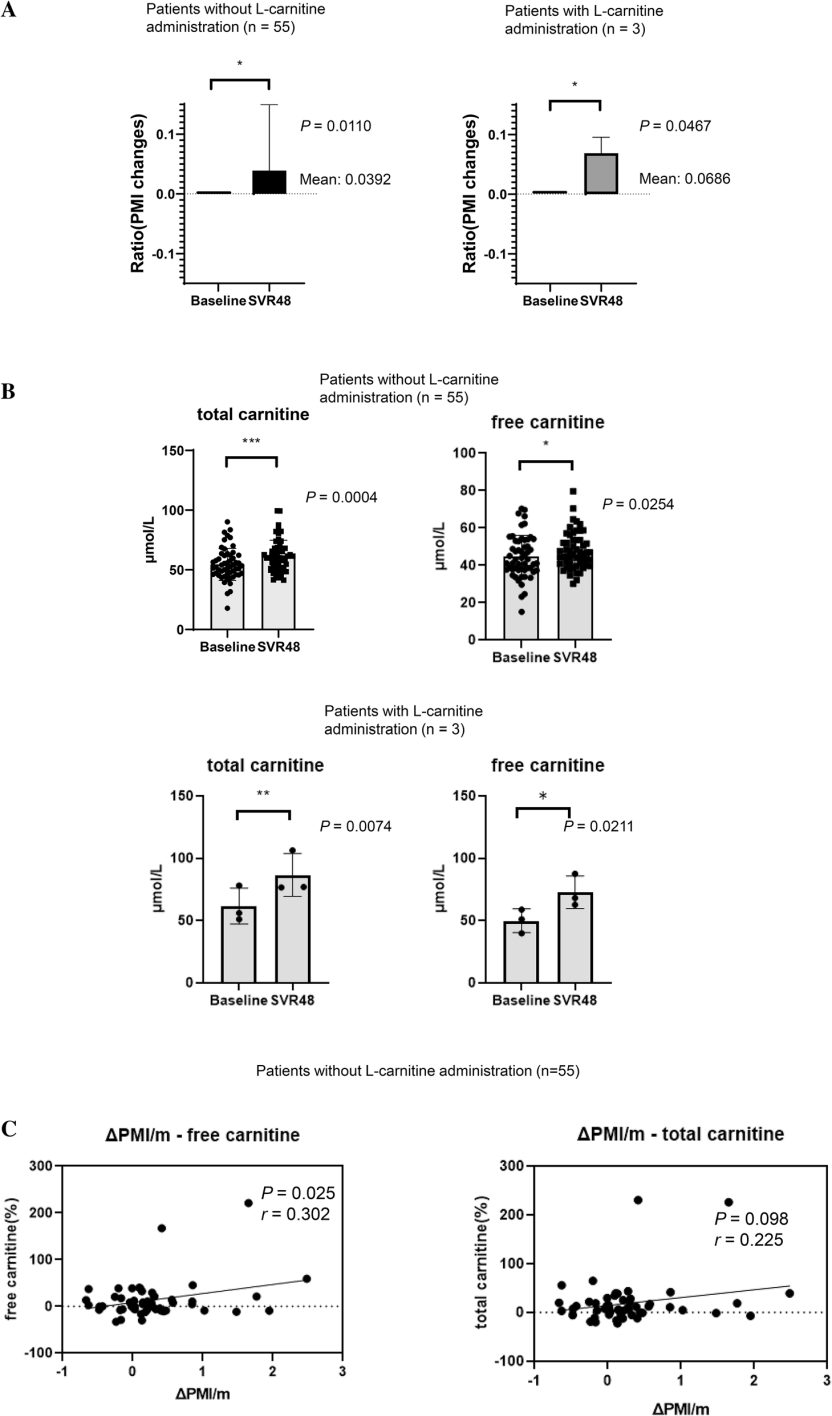
Comparison of data between patients with and without l-carnitine supplementation within the observational period. (**a**) Relative changes in the psoas muscle mass index (PMI) at SVR48 in patients with and without l-carnitine supplementation. (**b**) Changes in serum total and free carnitine levels between baseline and at SVR48 in patients with and without l-carnitine initiation. (**c**) Correlations between psoas muscle mass index (PMI) changes/month (ΔPMI/month) and changes in serum total/free creatinine in patients without l-carnitine initiation. *PMI* psoas muscle mass index. Data were analyzed using Spearman’s rank correlation.

Thus, increased free carnitine levels were clearly associated with an increased PMI after successful HCV eradication. Among patients with increased free carnitine levels after DAA therapy, three patients were administered l-carnitine (two for muscle cramps and one for hyperammonemia) during the observational period. Accordingly, we stratified patients according to l-carnitine administration. As shown in supplementary Table S1, the baseline patients’ characteristics were similar between patients with or without L-carnitine administration. As shown in Fig. 5a, even in patients without carnitine initiation during the observational period, the relative PMI values increased significantly (Fig. 5a) and serum total and free carnitine levels were significantly higher after successful HCV eradication by DAAs than at baseline (Fig. 5b). In addition, ΔPMI/month was significantly positively correlated with changes in free carnitine levels (Fig. 5c, r = 0.302, *P* = 0.025) in patients without l-carnitine administration.

All three patients treated with carnitine during the observational period exhibited increased PMI and free carnitine levels (Fig. 5).

## Discussion

Chronic liver disease is a major cause of secondary sarcopenia that is associated with poor prognosis in patients with chronic liver disease. Thus, proper management and prevention of sarcopenia are highly required in patients with chronic liver disease^17^. Recent reports have revealed that successful HCV eradication could increase skeletal muscle mass or suppress loss of skeletal muscle mass^10,11,20^. In this study, 70.7% of patients showed an increase in PMI and the mean relative PMI significantly increased after successful HCV eradication by DAAs. However, clinical factors examined in routine clinical practice, including age (supplementary Figure S1), did not show increased PMI values. Among the factors that were reported to be associated with skeletal muscle loss in patients with chronic liver disease, serum zinc as well as serum total and free carnitine levels increased significantly after DAA therapy. Additionally, only increased serum-free carnitine levels were significantly associated with increased PMI.

In patients with chronic liver disease, both suppression of skeletal muscle protein synthesis and promotion of skeletal muscle catabolism are thought to be involved in skeletal muscle mass loss. Vitamin D is involved in myoblast proliferation and differentiation^21,22^ and vitamin D deficiency is sometimes observed in patients with chronic liver disease. Thus, in patients with chronic liver disease, vitamin D deficiency is thought to be a cause of skeletal muscle mass loss. Similarly, IGF-1 deficiency, which is mainly produced in the liver via growth hormone stimulation, was observed in patients with chronic liver disease^23,24^. Because IGF-1 is associated with protein synthesis, it could result in skeletal muscle mass loss. Decreased liver function due to LC causes protein energy malnutrition; in such situations, BCAA, which has strong anabolic effects, is utilized in skeletal muscle mass^25^. Decreased BCAA levels cause dysfunctional protein synthesis. Furthermore, serum zinc levels were decreased in patients with chronic liver disease, and this decrease was significantly associated with sarcopenia^19^. Therefore, in these patients with chronic liver disease, decreased IGF-1, carnitine, BCAA, zinc, and vitamin D levels are important factors of skeletal muscle mass loss. In the present study, however, IGF-1, BCAA, and vitamin D levels did not change after successful HCV eradication, and changes in serum zinc levels were not associated with those in PMI. Thus, we suggest that increased PMI might not be associated with changes in serum IGF-1, BCAA, zinc, or vitamin D levels.

As shown in Fig. 2, serum total carnitine and free carnitine levels increased significantly after successful HCV eradication by DAAs. In addition, free carnitine alone was significantly and positively correlated with ΔPMI/month. Thus, increased serum-free carnitine level was associated with increased PMI after successful HCV eradication by DAAs. Carnitine is an essential nutrient that is mainly involved in fatty acid metabolism^26^. Total carnitine comprises free carnitine and all acylcarnitines^27^ and most of carnitine exists as free carnitine^28^. Blood free carnitine less than < 20 μmol/L is defined as carnitine deficiency. Although carnitine is absorbed mainly from dietary intake, approximately 25% of carnitine is generated in the liver and kidney^29^. Thus, in patients with chronic liver or kidney disease, secondary carnitine deficiency is usually observed^26,30^. The effect of carnitine deficiency could be predicted based on patients with primary carnitine deficiency, who showed hepatic steatosis, hyperammonemia, and skeletal myopathy^31,32^. Thus, carnitine is thought to be involved in muscle conditions. Recently, we have reported that carnitine supplementation in patients with LC could suppress skeletal muscle mass loss^18^. The mechanisms by which carnitine suppresses skeletal muscle loss and by which increased serum-free carnitine increases PMI have not been fully clarified. However, several hypotheses have been proposed. Carnitine exists at high concentrations in the muscle^33^; thus, skeletal muscle might be strongly affected by a carnitine deficiency.

Hyperammonemia was observed in patients with primary carnitine deficiency and could cause muscle atrophy via upregulation of myostatin, which suppressed muscle synthesis^34^. We and other studies have revealed that carnitine supplementation could restore hyperammonemia^18^. In addition, based on in vitro and clinical analyses, we have previously reported that carnitine supplementation could suppress inflammatory parameters and oxidative stress in liver disease^18,35^. Since inflammation and increased oxidative stress could cause skeletal muscle loss^36^, the anti-inflammatory and anti-oxidative stress effects of carnitine might contribute to increased PMI after successful HCV eradication by DAAs.

The mechanisms underlying increased carnitine levels after successful HCV eradication are not clear. However, since increased carnitine levels are significantly correlated with increased serum albumin levels (Supplementary Figure S2), restoration of liver function after successful HCV eradication might be involved. Further analyses are required to clarify this association.

Additionally, in all three patients with l-carnitine supplementation, a significant increase in PMI was observed. Beneficial effects of L-carnitine supplementation in patients with chronic liver disease, including improvement of impaired brain function^37^, muscle cramp^38^, and restoring hyperammonemia^18^, have been reported. This study results might highlight that in patients with low skeletal muscle mass even after DAA therapy, l-carnitine supplementation with is a potential therapeutic option.

This study has several limitations. First, this was a single-center retrospective study, and the number of patients was limited. In addition, data of several factors, including serum ammonia, were lacking owing to the retrospective nature of the study. In this study, we could not show the baseline factors predicting the increased skeletal muscle mass after successful HCV eradication. Therefore, a large multicenter prospective study is required.

In conclusion, increased skeletal muscle mass after successful HCV eradication by DAAs was significantly associated with increased serum-free carnitine levels. This finding might suggest that l-carnitine supplementation may be beneficial in patients with low skeletal muscle mass after DAA therapy.

## Materials and methods

### Patients and study design

HCV-infected patients who were treated with DAAs and achieved SVR between October 2014 and November 2019 at Hokkaido University Hospital were retrospectively screened. Patients were included if they had paired computed tomography (CT) examinations at baseline (within 6 months before DAA initiation) and at 12 months or longer after completion of treatment, proper clinical information, and preserved serum samples obtained at baseline and at 12 months after treatment completion (SVR48). Patients were excluded if they could not achieve SVR, did not have paired CT examinations, did not have preserved serum samples at both baseline and SVR48, had insufficient clinical data, had other liver diseases, had a history of liver transplantation or hemodialysis, or were co-infected with human immunodeficiency virus or hepatitis B virus.

Clinical data of patients, CT results of skeletal muscle mass analysis, and preserved serum samples at baseline and SVR48 were collected. In addition, concomitant drugs use was monitored during the observational period, including l-carnitine, which inhibits loss of skeletal muscle mass.

Presence of LC was diagnosed by liver biopsy, FibroScan data, radiologic findings, and laboratory data^18^. Preserved serum was used to analyze serum levels of 25(OH)-vitamin D, total/free carnitine, zinc, BCAA, and IGF-1, which have been reported to be associated with skeletal muscle loss in patients with liver disease^18,19^. Serum IGF1 and 25(OH)-vitamin D were analyzed by the immunoradiometric assay, as previously described^18^. Serum concentrations of total and free carnitine, zinc, and BCAA levels were analyzed at the clinical laboratory of SRL (Tokyo, Japan).

The study protocol was approved by the Ethics Committee of Hokkaido University Hospital (approval numbers: 016-0021 and 016-0397). In addition, the study protocol conformed to the ethical guidelines of the Declaration of Helsinki. All patients included in the study provided written informed consent prior to participation.

### Calculation of skeletal muscle mass

Skeletal muscle mass was evaluated using the psoas muscle mass index (PMI), as previously described^39,40^. Briefly, the PMI was calculated from paired CT as the sum of cross-sectional areas at the L3 level of the right and left psoas muscle masses by manual tracing; this value was divided by height squared^18,39,40^. The PMI was evaluated at baseline and at ≥ 12 months after treatment completion.

Additionally, monthly changes in the PMI (ΔPMI/month) were evaluated as follows: (psoas muscle area on CT scan at ≥ 12 months after DAA completion – psoas muscle area on the baseline CT scan)/psoas muscle area on the baseline CT scan]/interval between CT scans (months)^11,39,40^.

### Statistical analysis

Continuous variables were analyzed using the Mann–Whitney U test or paired *t*-test. Categorical variables were analyzed using Fisher’s exact test. Relationships between two variables were assessed using the Spearman’s rank correlation. All statistical analyses were performed using Prism 7.03 (GraphPad Software, Inc., La Jolla, CA, USA). All *P*-values were two-tailed, and *P* < 0.05 indicated statistical significance.

## Supplementary Information

Supplementary Information 1.

Supplementary Information 2.

Supplementary Information 3.

Supplementary Information 4.

## Data Availability

The data that support the findings of this study are available from the corresponding author upon reasonable request.
