# Supplementary material for: Possible correlation between increased serum free carnitine levels and increased skeletal muscle mass following HCV eradication by direct acting antivirals
Source: Sci Rep. 2021 Aug 16;11:16616. doi: 10.1038/s41598-021-96203-z (PMC8368156; doi:10.1038/s41598-021-96203-z)
Supplement: Supplementary file 1 — Supplementary Information 1. [file 41598_2021_96203_MOESM1_ESM.docx]

**Supplementary Table S1. Comparison between patients with or without L-carnitine administration**

|  | L-carnitine p. o. (+) | L-carnitine p. o. (-) | P value |
| --- | --- | --- | --- |
| Number | 3 | 55 |  |
| Age (years)^a^ | 73.0(61-75) | 74.0(43-90) | 0.7703 |
| Sex (male/female) | 1/2 | 27/28 | ＞0.9999 |
| HCV-RNA (log IU/mL) ^a^ | 6.2(6.0-6.6) | 6.2(3.7-7.2) | 0.5183 |
| HCV Cerotype (1/2/3/4) | 3/0/0/0 | 44/10/0/1 | 0.691 |
| DCV/ASV, SOF/LDV, SOF/RBV, OBV/PTV/r, others | 2/1/0/0/0 | 11/18/9/6/11 | 0.3819 |
| LC/nonLC | 2/1 | 26/29 | 0.6053 |
| Platelet count (×10^4^) ^a^ | 7.5(6.7-13.6) | 11.9(4.3-35.9) | 0.2834 |
| Albumin (g/dL) ^a^ | 3.5(2.7-3.9) | 3.7(2.7-4.8) | 0.2992 |
| AST (IU/L) ^a^ | 62(53-79) | 44(17-155) | 0.4115 |
| ALT (IU/L) ^a^ | 45(35-114) | 35(14-128) | 0.1985 |
| FIB-4 index ^a^ | 8.8(3.2-9.2) | 5.0(0.70-12.0) | 0.3012 |
| AFP (ng/mL) ^a^ | 8.7(5.3-12.9) | 6.6(1.5-83.9) | 0.6892 |
| Creatine(mg/dl) | 0.63(0.61-0.92) | 0.80(0.41-1.81) | 0.4922 |
| Diabetes mellitus n (%) | 0(0) | 11(20.0) | N/A |
| Previous treatment (yes/ no) | 1/2 | 18/37 | ＞0.9999 |
| PMI (cm^2^/m^2^) ^a^ | 4.59(4.53-5.35) | 4.23(2.08-8.46) | 0.4417 |
| Body weight (kg) | 58.0(44-61) | 59.0(32.0-102.0) | 0.5777 |
| BMI(kg/m²) | 22.6(19.8-26.8) | 22.5(15.0-34.1) | 0.9504 |

Abbreviations: HCV, Hepatitis C virus; AST, aspartate aminotransferase; ALT, alanine aminotransferase; FIB-4, fibrosis 4. ^a^Data are shown as median (range) values. p.o, per os; *Statistically significant difference, P <0.05
