# Supplementary figures and images for: Possible correlation between increased serum free carnitine levels and increased skeletal muscle mass following HCV eradication by direct acting antivirals
Source: Sci Rep. 2021 Aug 16;11:16616. doi: 10.1038/s41598-021-96203-z (PMC8368156; doi:10.1038/s41598-021-96203-z)

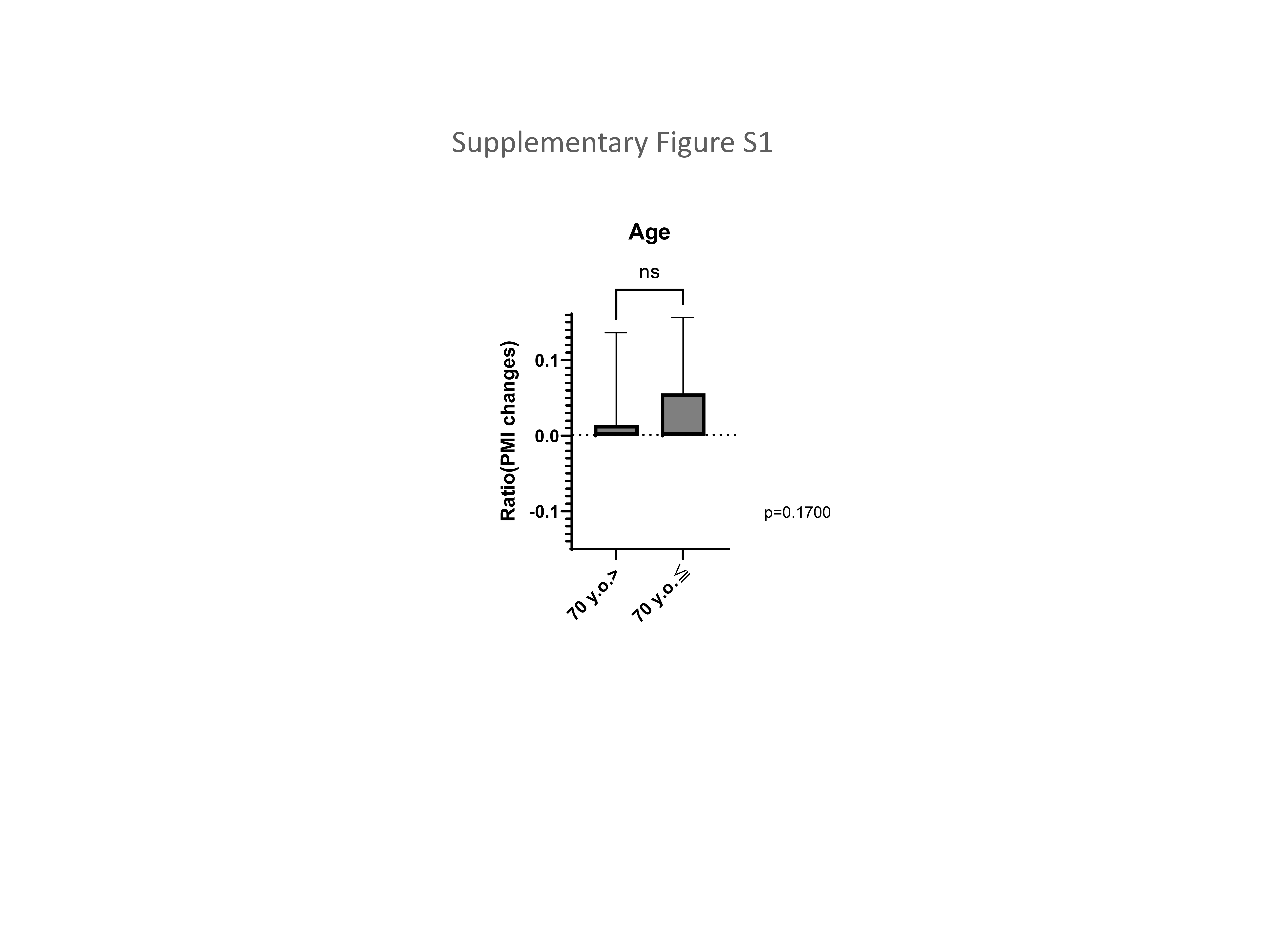

Supplement: Supplementary file 2 — Supplementary Information 2. [file 41598_2021_96203_MOESM2_ESM.tif]

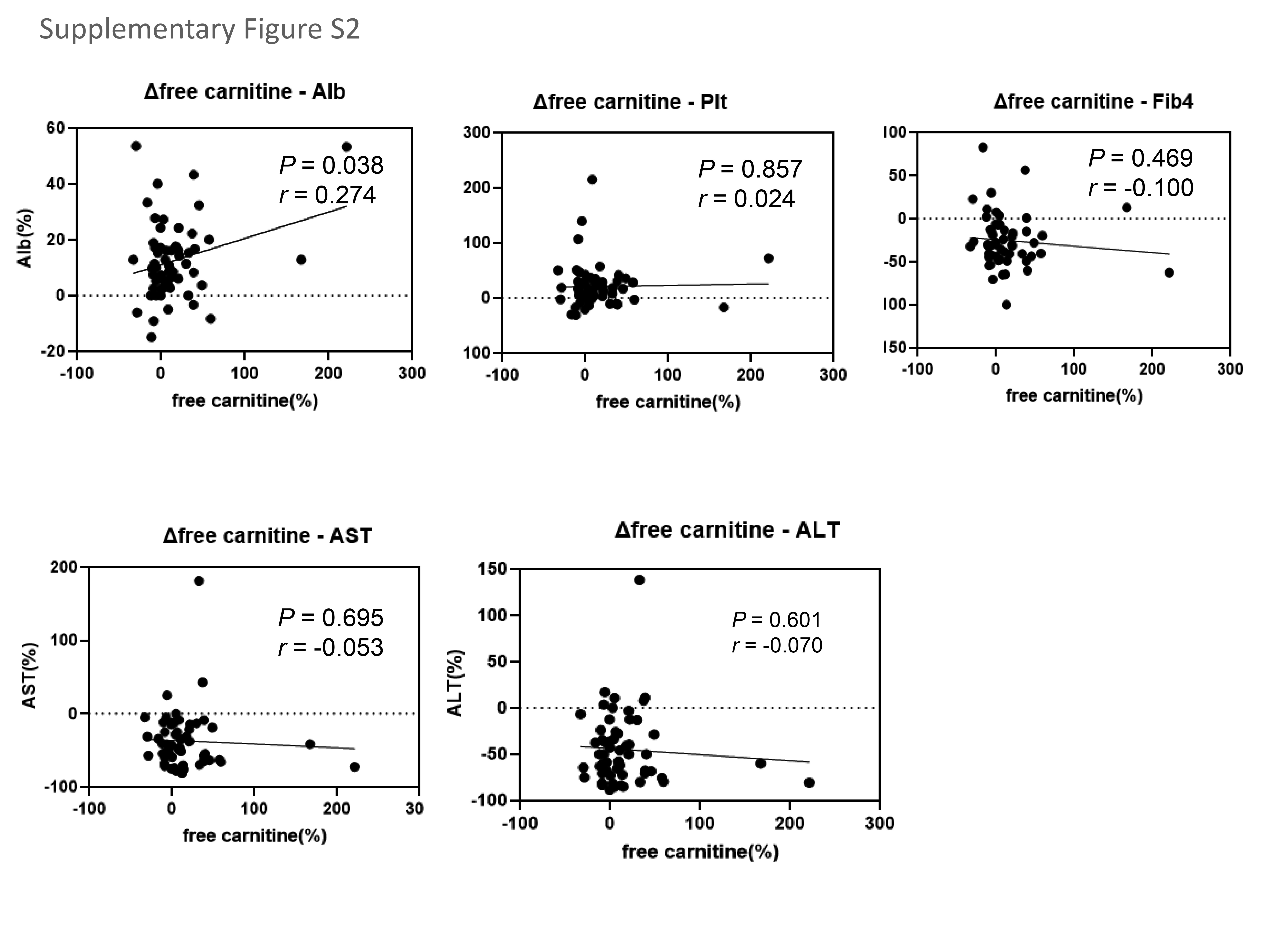

Supplement: Supplementary file 3 — Supplementary Information 3. [file 41598_2021_96203_MOESM3_ESM.tif]
