# Supplementary material for: Possible correlation between increased serum free carnitine levels and increased skeletal muscle mass following HCV eradication by direct acting antivirals
Source: Sci Rep. 2021 Aug 16;11:16616. doi: 10.1038/s41598-021-96203-z (PMC8368156; doi:10.1038/s41598-021-96203-z)
Supplement: Supplementary file 4 — Supplementary Information 4. [file 41598_2021_96203_MOESM4_ESM.docx]

**Supplementary Material**

**Possible correlation between increased serum free carnitine levels and increased skeletal muscle mass following HCV eradication by direct acting antivirals**

Yoshimasa Tokuchi^1^, Goki Suda¹^*^, Megumi Kimura¹, Osamu Maehara², Takashi Kitagataya¹, Kubo Akinori^1^, Sonoe Yoshida^1^, Qingjie Fu¹, Yang Zijian¹, Shunichi Hosoda¹, Masatsugu Ohara¹, Ren Yamada¹, Kazuharu Suzuki¹, Naoki Kawagishi¹, Masato Nakai¹, Takuya Sho¹, Mitsuteru Natsuizaka¹, Kenichi Morikawa¹, Koji Ogawa^1^, Shunsuke Ohnishi², Naoya Sakamoto¹

**Table of Contents:**

**Supplementary Table S1. Comparison between patients with or without L-carnitine administration**

**Supplementary Figure S1.**

**Comparison of relative changes in skeletal muscle mass after successful HCV eradication by direct-acting antiviral agents (DAAs) at SVR48 according to age.**

SVR48; sustained virological response at 48 weeks after the completion of treatment

PMI; psoas muscle mass index

Data are shown as means ± standard deviation (SD).

**Supplementary Figure S2.**

**Correlations between changes in serum free carnitine levels and changes in serum albumin, platelet count, Fib4 index, aspartate aminotransferase, and alanine aminotransferase**

Alb: albumin

Plt: platelet count

AST: aspartate aminotransferase

ALT: alanine aminotransferase

Data were analyzed using Spearman’s rank correlation.
